# Supplementary material for: Successive Pandemic Waves with Different Virulent Strains and the Effects of Vaccination for SARS-CoV-2
Source: Vaccines (Basel). 2022 Feb 22;10(3):343. doi: 10.3390/vaccines10030343 (PMC8952817; doi:10.3390/vaccines10030343)
Supplement: Supplementary file 1 [file vaccines-10-00343-s001.zip › vaccines-1526588-supplementary/Supplementary File - Castro-e-Silva et al..pdf]

vaccines

MDPI

Supplementary File

Successive pandemic waves with more virulent strains, and the effects of vaccination for SARS-CoV-2

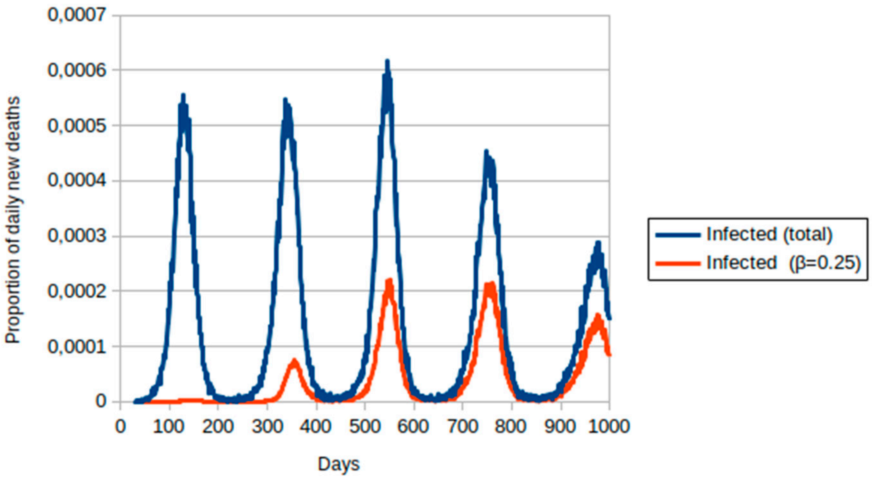

Figure S1. ABM-SIR dynamic of COVID-19 evolution along 1,000 days, adjusted for the initial population of ~ 8.75×10^5 individuals. Only the proportion of new dead individuals is shown, represented by the blue line. The proportion those among them who died by the most lethal variant is represented by the red line.

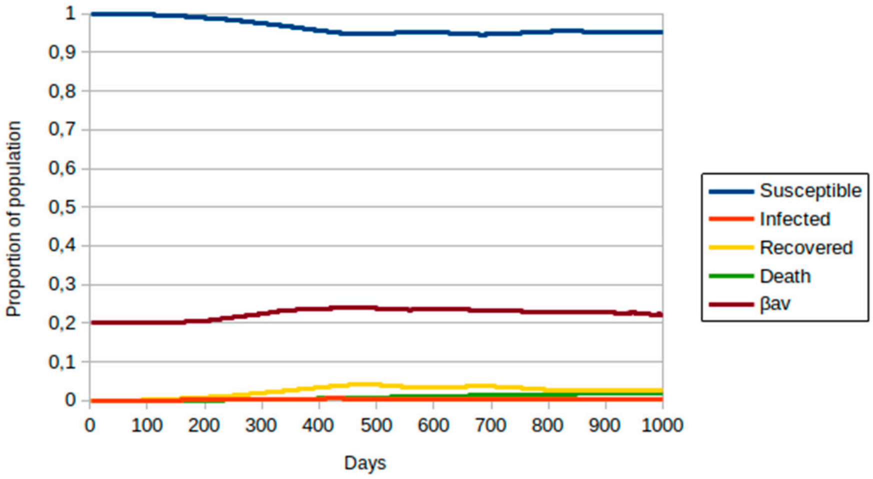

Figure S2. ABM-SIR dynamic of COVID-19 evolution along 1,000 days for social isolation of 70%, adjusted for the initial population of ~ 8.75×10^5 individuals. There is no vaccination. Each color represents a population group, defined in the legend. The value of β_{av} is the average over all infected people.

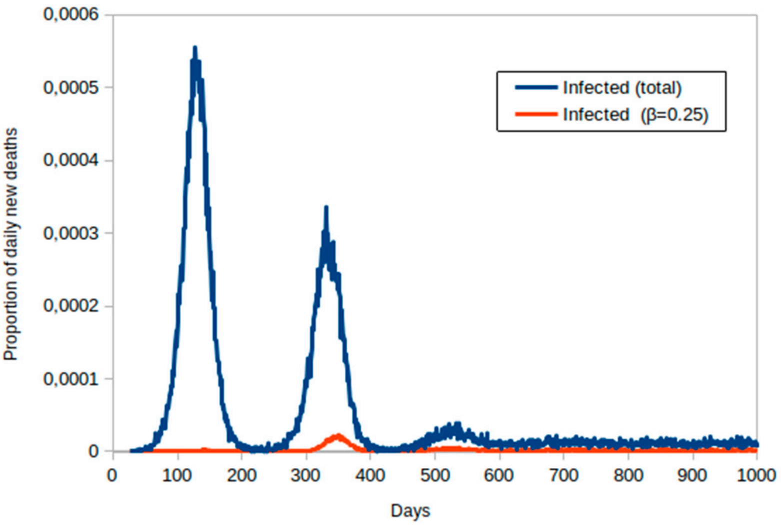

Figure S3. ABM-SIR dynamic of COVID-19 evolution along 1,000 days for social isolation of 40%, adjusted for the initial population of ~ 8.75 × 10^5 individuals. Only the proportion of new dead individuals is shown, represented by the blue line. The proportion those among them who died by the most lethal variant is represented by the red line. Vaccination rate is 1/200 of susceptible or recovered individuals per day.

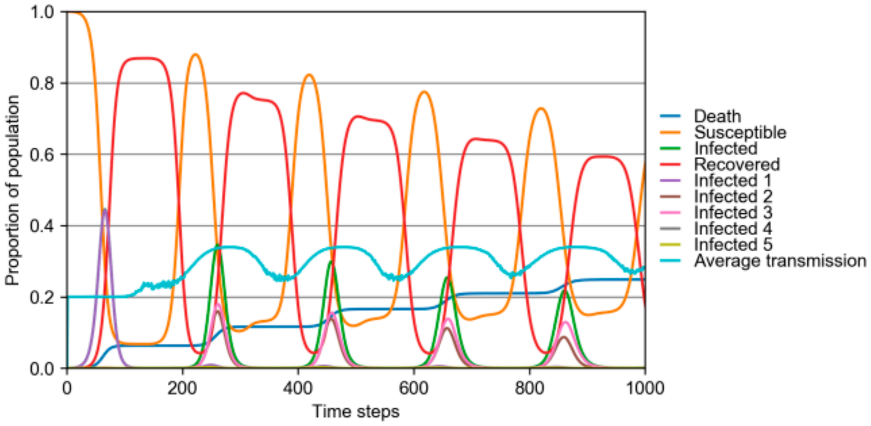

Figure S4. ABM-SIR dynamic of COVID-19 evolution along 1,000 days, adjusted for the initial population of 1M, with variants entering via travelers. The social distancing is 45%. The values of β and mortality for infected can be seen in Table 1. In this scenario, all recovered lost their immunity after 120 days.
